# Friction behavior of a microstructured polymer surface inspired by snake skin

**DOI:** 10.3762/bjnano.5.8

**Published:** 2014-01-24

**Authors:** Martina J Baum, Lars Heepe, Stanislav N Gorb

**Affiliations:** 1 Functional Morphology and Biomechanics, Zoological Institute, Kiel University, Am Botanischen Garten 1–9, Kiel 24098, Germany

**Keywords:** fast Fourier transformation, friction, polymer, snake inspired, stick-slip

## Abstract

The aim of this study was to understand the influence of microstructures found on ventral scales of the biological model, *Lampropeltis getula californiae*, the California King Snake, on the friction behavior. For this purpose, we compared snake-inspired anisotropic microstructured surfaces to other microstructured surfaces with isotropic and anisotropic geometry. To exclude that the friction measurements were influenced by physico-chemical variations, all friction measurements were performed on the same epoxy polymer. For frictional measurements a microtribometer was used. Original data were processed by fast Fourier transformation (FFT) with a zero frequency related to the average friction and other peaks resulting from periodic stick-slip behavior. The data showed that the specific ventral surface ornamentation of snakes does not only reduce the frictional coefficient and generate anisotropic frictional properties, but also reduces stick-slip vibrations during sliding, which might be an adaptation to reduce wear. Based on this extensive comparative study of different microstructured polymer samples, it was experimentally demonstrated that the friction-induced stick-slip behavior does not solely depend on the frictional coefficient of the contact pair.

## Introduction

The absence of extremities in snakes has strong tribological consequences for the material of their skin. The ventral body side of the snake is in continuous contact with the substrate. Therefore ventral scales must have optimized frictional properties. In order to generate propulsion during locomotion high friction and to slide along the substrate low friction must be generated [1]. Additionally, a minimum abrasion rate is necessary to enable long lasting optimal frictional properties [1]. Thus, to facilitate effective locomotion, the ventral body surface needs to possess anisotropic frictional properties, which can originate from macroscopic structures [2–3] such as the overlapping scales. The arrangement of scales provides the possibility of interlocking between their edges and asperities of the substrate. Also microscopic structures of the skin scales, so called microornamentation [1,4–13], and specific adaptations of the material architecture of the skin, like highly ordered embedded fibers [14], which can potentially influence material properties [15–16], might contribute to the frictional anisotropy.

**Figure 1:**
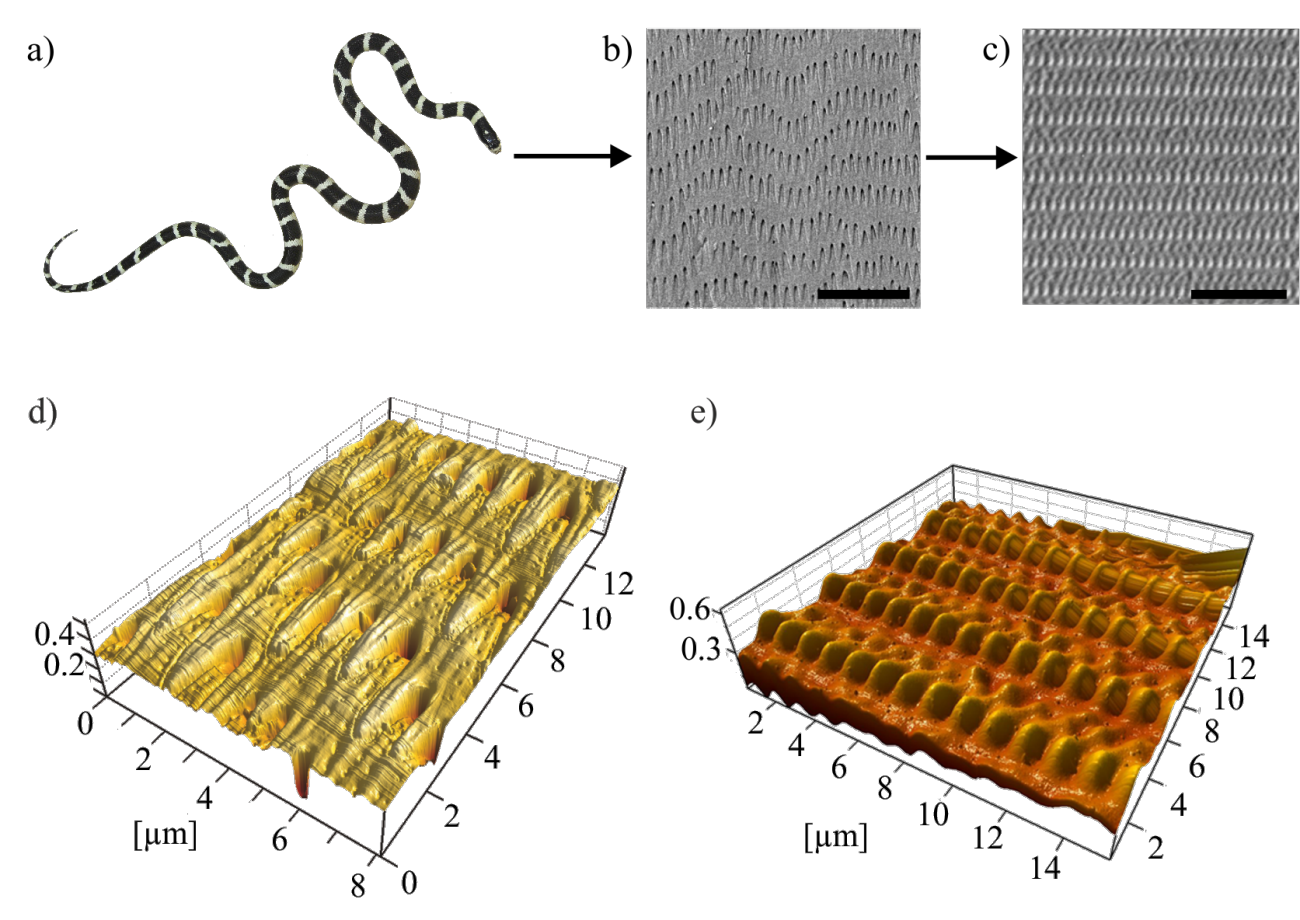
From snake skin to SIMPS. a) Photograph of *L. g. californiae* , the California King Snake; b) SEM micrograph of a ventral scale of *L. g. californiae*; c) SEM micrograph of the snake-inspired microstructured polymer surface – SIMPS. Scale bars: 10 µm. 3-dimensional image of d) snake skin from *L. g. californiae* and e) SIMPS based on AFM data.

The role of microornamentation in frictional properties of the snake skin was extensively examined [2–3,9,11–12]. We previously showed a strong influence of the stiffness of the underlying layers of the epidermis on the anisotropic frictional properties of the skin [17]. This finding demonstrates “snake skin” as a highly complex frictional system with numerous variables influencing its frictional properties. The surface of the ventral scale of the snake *Lampropeltis getula californiae* (Figure 1a) was previously examined in detail by scanning electron microscope (SEM) (Figure 1b). Based on this morphological data [17], snake-inspired microstructured polymer surfaces (SIMPS) [18] were developed (Figure 1c). Such an implementation of the surface geometry, similar to biological microstructures of the snake, into a mechanically and chemically well-defined polymeric material, epoxy resin [19], enabled us a comparable and reproducible investigation of the influence of the surface microstructure on the frictional properties.

In order to gain insight in the influence of the snake-inspired anisotropic surface topography, additional surface topographies were investigated. The frictional coefficient of these surfaces and their stick-slip behavior were compared with those of SIMPS. The phenomenon of stick-slip is known to influence friction as well as abrasion and thus is important for technical contact surfaces. Stick-slip motion, the quasi-periodic “sticking” and “slipping” of the contacting surfaces under relative motion, can be often observed on dry, non-lubricated contacts [20–24].

The dimensions, in which this phenomenon occurs, can vary from macroscopic to atomic ones. The underlying physical effects range from the interlocking of surface asperities to van-der-Waals force [22–26]. Stick-slip is hard to describe, because it is affected by multiple geometrical and physical parameters of both frictional partners influencing the resonance frequency and its dampening. In most technical systems, the stick-slip phenomenon is unwanted, because it leads to vibrations, sometimes with acoustic emissions, to an increase in abrasion and to stronger energy dissipation, to an increase in maintenance costs of industrial facilities due to (1) the higher need of lubricants, (2) the replacement costs of machine parts, and (3) the loss of productivity due to maintenance. However, the presence of the stick-slip phenomenon might be also welcome in a small number of applications, like in playing the violin by moving the bow over the strings and inducing stick-slip-based vibrations of the strings [23,25–26].

In order to describe the frictional behavior in our frictional system, we have chosen an experimental setup with a limited number of variables. Using a microtribometer frictional properties were characterized by measuring tangential and normal force for the relative motion of a smooth glass ball (diameter 1 mm) brought into contact with nominal flat surfaces. The frictional counterpart (substrate) was kept constant, enabling us to investigate the influence of different surface topographies of polymer samples on the stick-slip phenomenon and on the frictional behavior in general. The different types of samples bear microstructures in comparable dimensions to those of the snake skin. Each selected type of microstructure is used to investigate the influence of certain features of snake scales responsible for specific frictional behavior.

## Results

In order to characterize the influence of surface topography on frictional properties frictional measurements on differently microstructured polymer surfaces were performed. In this study we used randomly rough surfaces (RRS) with isotropic roughness. By contrast, periodical groove-like polymer surface (PGMS) consisted of equal lines and spaces with well-defined pitch lengths. The geometry of the microstructures on polymer molds of *L. g. californiae* (PMLG) from ventral scales of the snake are regular tooth-like shaped and caudally oriented (parallel to the snake’s body axis). The microstructure of the snake-inspired microstructured polymer surface (SIMPS) can be abstracted as a geometrical combination of parallel lines (the denticulations), which are periodically interrupted by the elevated tips. This microstructure possesses similar shapes and dimensions of those of the biological model, the microornamentation of the ventral scales of *L. g. californiae*. Detailed information on the investigated surfaces is listed in Table 1.

**Table 1:** Surface roughness (Ra) of all investigated polymer surfaces. "On line" roughness was measured on top of the line, along the microstructure. λ: pitch dimension. SD: standard deviation.

Sample			*R*_a_ [µm]	SD

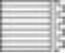	Periodic groove-like microstructure	PGMS - λ: 5 µm	0.18	0.02
	Periodic groove-like microstructure	PGMS - λ: 25 µm	4.95	0.37
	Periodic groove-like microstructure	PGMS - λ: 50 µm	21.75	0.26
	Periodic groove-like microstructure	PGMS - λ: 100 µm	42.50	1.47
	Periodic groove-like microstructure	PGMS - on line	0.03	0.01

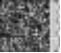	Randomly rough surface	RRS - 0.3 µm	0.23	0.00
	Randomly rough surface	RRS - 1 µm	0.41	0.01
	Randomly rough surface	RRS - 3 µm	1.11	0.11
	Randomly rough surface	RRS - 9 µm	2.39	0.07
	Randomly rough surface	RRS - 12 µm	7.64	0.13

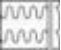	Polymeric mold of *L. g. californiae*	PMLG	0.09	0.04

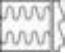	Snake-inspired microstructured surface	SIMPS	0.10	0.13

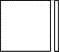	Smooth surface	Smooth surface	0.02	0.01

### Stick-slip behavior of periodical groove-like polymer surface (PGMS)

The results obtained of the FFT offer the possibility to determine the mean frictional coefficient based on the signal’s bias, which is determined as the amplitude of the zero-frequency. For the PGMS, these results are listed in Table 2.

**Table 2:** Mean frictional coefficients (µ) of periodical groove-like polymer surface – PGMS determined by the zero-frequency of the FFTs and standard deviation (SD). λ: pitch dimension.

Sample	Frictional coefficient in direction of measurement relative to orientation of microstructure:			
	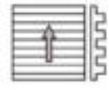 perpendicular		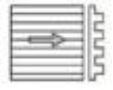 parallel	
	µ	SD	µ	SD

PGMS - λ: 5 µm	0.30	0.01	0.23	0.02
PGMS - λ: 25 µm	0.16	0.01	0.21	0.01
PGMS - λ: 50 µm	0.18	0.01	0.22	0.02
PGMS - λ: 100 µm	0.24	0.01	0.19	0.02

**Figure 2:**
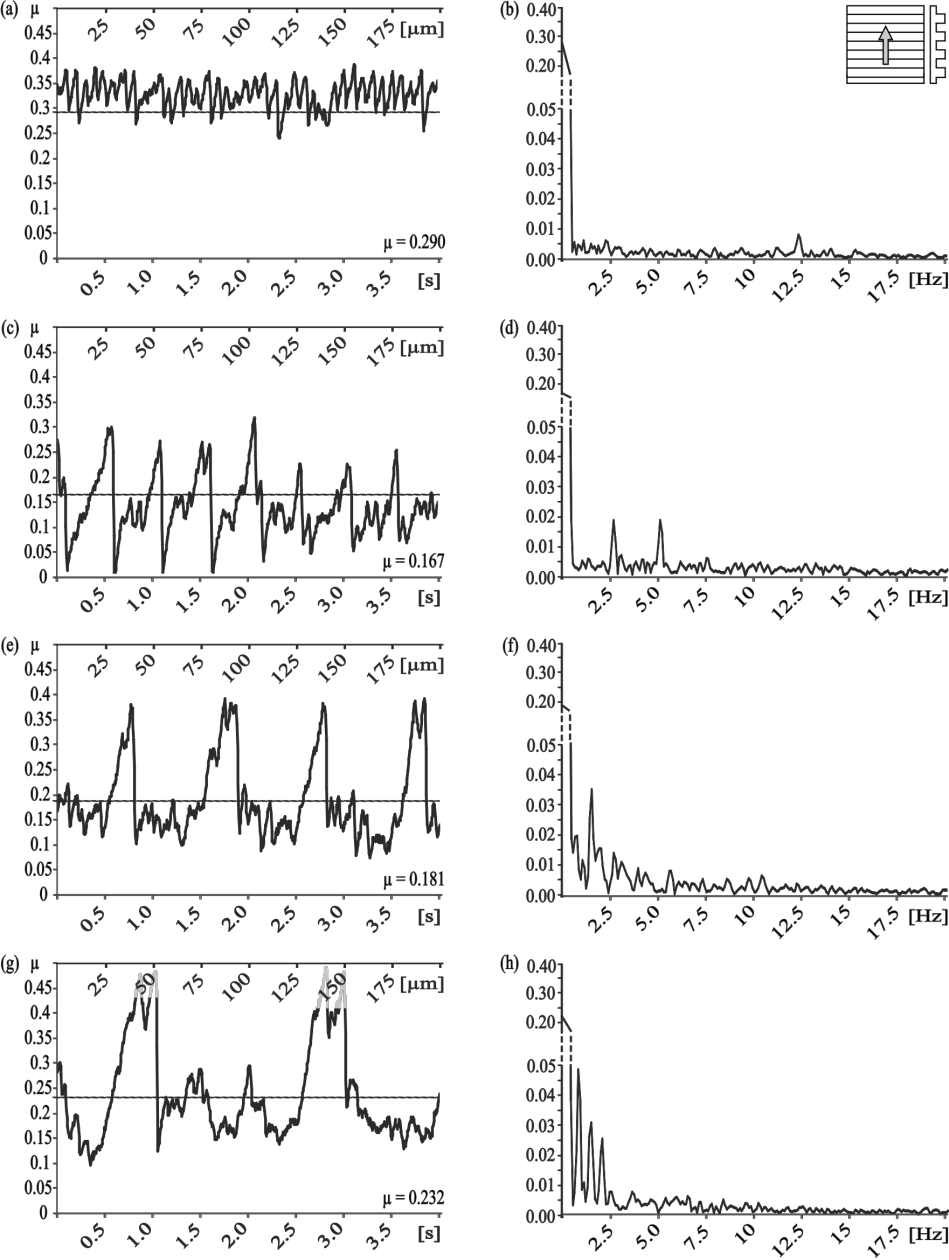
Results of frictional measurements on periodical groove-like polymer surface – PGMS perpendicular to the orientation of the microstructure. Left column, frictional signal in the spatial/time domain. Right column, frictional signal in the frequency domain after FFT; the ordinate shows the single-sided amplitude spectrum – SSAS. PGMS pitch dimension: a,b) 5 µm, c,d) 25 µm, e,f) 50 µm and g,h) 100 µm.

Frictional measurements performed perpendicular to the orientation of the microstructures of the PGMSs showed a wide variety of frictional responses (Figure 2). The surface with a pitch dimension of 5 µm shows a dominant frequency at 12.2 Hz, which, at the used speed of 50 µm/s, corresponds to a wavelength of 4.1 µm (Figure 2b). A comparable correlation between the microstructure’s pitch dimension and the dominating frequency can also be found for all other PGMS (Figure 2d,f,h). Superimposed harmonic oscillations can be found for pitch dimensions of 25 µm and 100 µm (Figure 2d,h).

**Figure 3:**
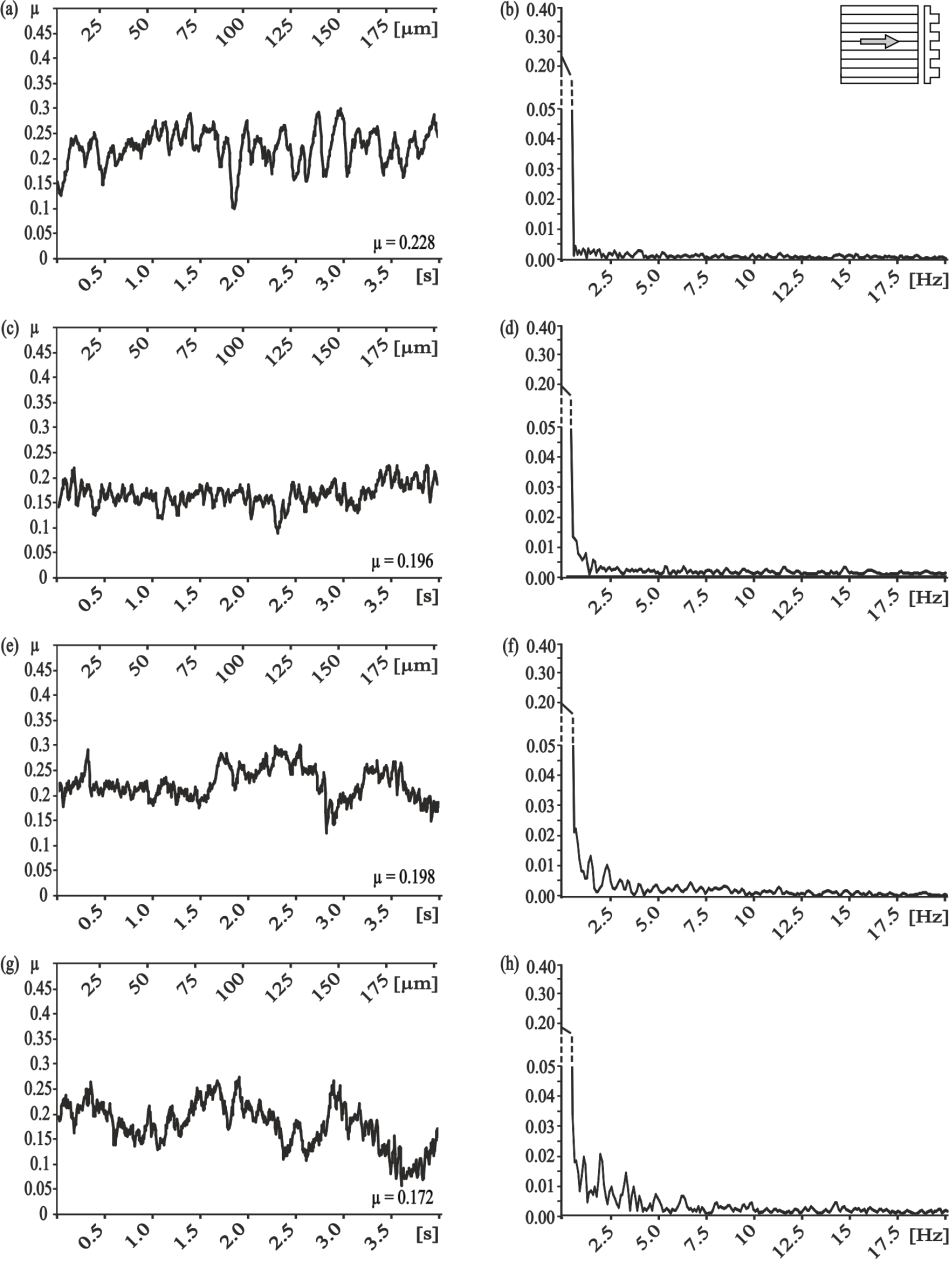
Results of frictional measurements on periodical groove-like polymer surface – PGMS parallel to the orientation of the microstructure. Left column, frictional signal in the spatial/time domain. Right column, frictional signal in the frequency domain after FFT; the ordinate shows the single-sided amplitude spectrum – SSAS. PGMS pitch dimension: a,b) 5 µm, c,d) 25 µm, e,f) 50 µm, g,h) 100 µm.

With the measurement direction rotated by 90°, we found completely different results for the frequency analysis (Figure 3). For the smallest and the second smallest pitch dimensions (5 µm and 25 µm, respectively) no dominant frequency was found (Figure 3b,d). A correlation to the pitch dimension was found for the PGMS with a structural wavelength of 50 µm, nevertheless the amplitude of the frequency spectrum was quite low (Figure 3f). Similar low amplitudes were found for the PGMS 100 µm, but the observed frequencies do not directly correlate with the microstructure dimension (Figure 3h).

Comparison of the frictional behavior in different sliding directions within each dimension of pitches, showed a strong frictional anisotropy and strong variation in the occurrence of stick-slip induced vibrations, especially at pitch dimensions of 5 µm and 25 µm. For the smallest dimension of PGMS, less vibrations and a lower frictional coefficient occurred at parallel direction of measurement, if compared to the perpendicular direction (Table 2; Figure 2b; 3b). For the PGMS with a structural wavelength of 25 µm, the vibration frequency in the parallel direction was also reduced, but the frictional coefficient in this direction was higher, than in the perpendicular direction (Table 2; Figure 2d; 3d). The results obtained on the other PGMS dimensions (50 µm and 100 µm) do not allow a clear statement on the stick-slip behavior, but a variation in the frictional coefficient between the directions of measurement were found as well (Table 2; Figure 2f,h; Figure 3f,h).

### Stick-slip behavior of randomly-rough surfaces (RRS) with different grain size

**Figure 4:**
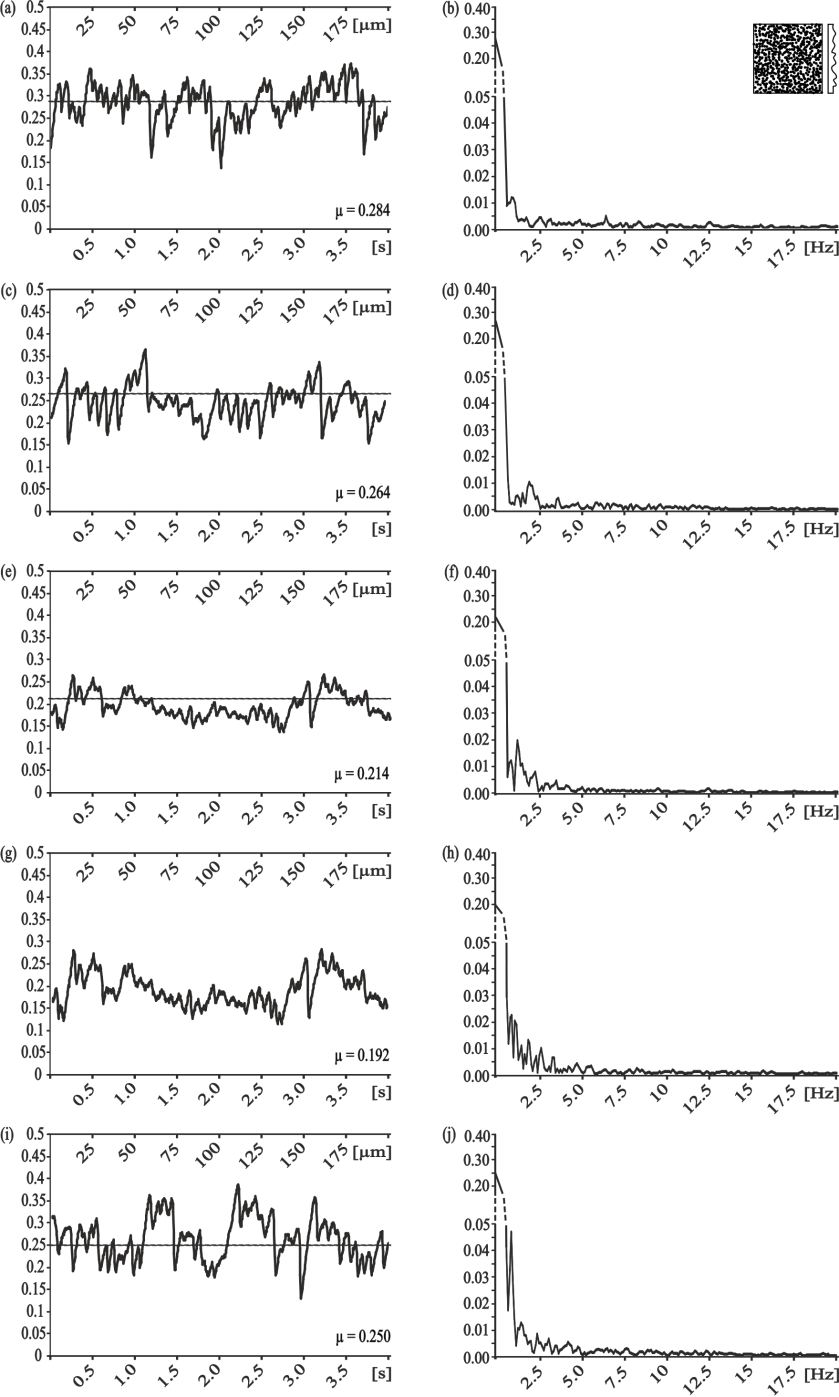
Results of frictional measurements on randomly-rough surfaces – RRS. Left column, frictional signal in the spatial/time domain. Right column, frictional signal in the frequency domain after FFT; the ordinate shows the single-sided amplitude spectrum - SSAS. Grain size of RRS: a,b) 0.3 µm, c,d) 1 µm, e,f) 3 µm, g,h) 9 µm, i,j) 12 µm.

The frictional measurements on RRS showed a strong influence of the surface roughness on the frictional coefficient. A minimum in friction was measured on a surface with grain size of 9 µm (Table 3). In the frequency domain, the amplitude of dominant frequencies are medium (maximal amplitude: 0.015–0.025) (Figure 4b,d) for the smaller grain size, and strong (Figure 4f,h) to very strong (Figure 4j) (maximal amplitude: >0.025) for the bigger grain sizes.

**Table 3:** Mean frictional coefficients of randomly-rough surfaces – RRS determined by zero-frequency of the FFTs curve.

Sample	Frictional coefficient	
	µ	SD

RRS - 0.3 µm	0.27	0.01
RRS - 1 µm	0.26	0.00
RRS - 3 µm	0.20	0.01
RRS - 9 µm	0.19	0.00
RRS - 12 µm	0.24	0.01

### Stick-slip behavior of epoxy molds of ventral snake scales (PMLG)

**Figure 5:**
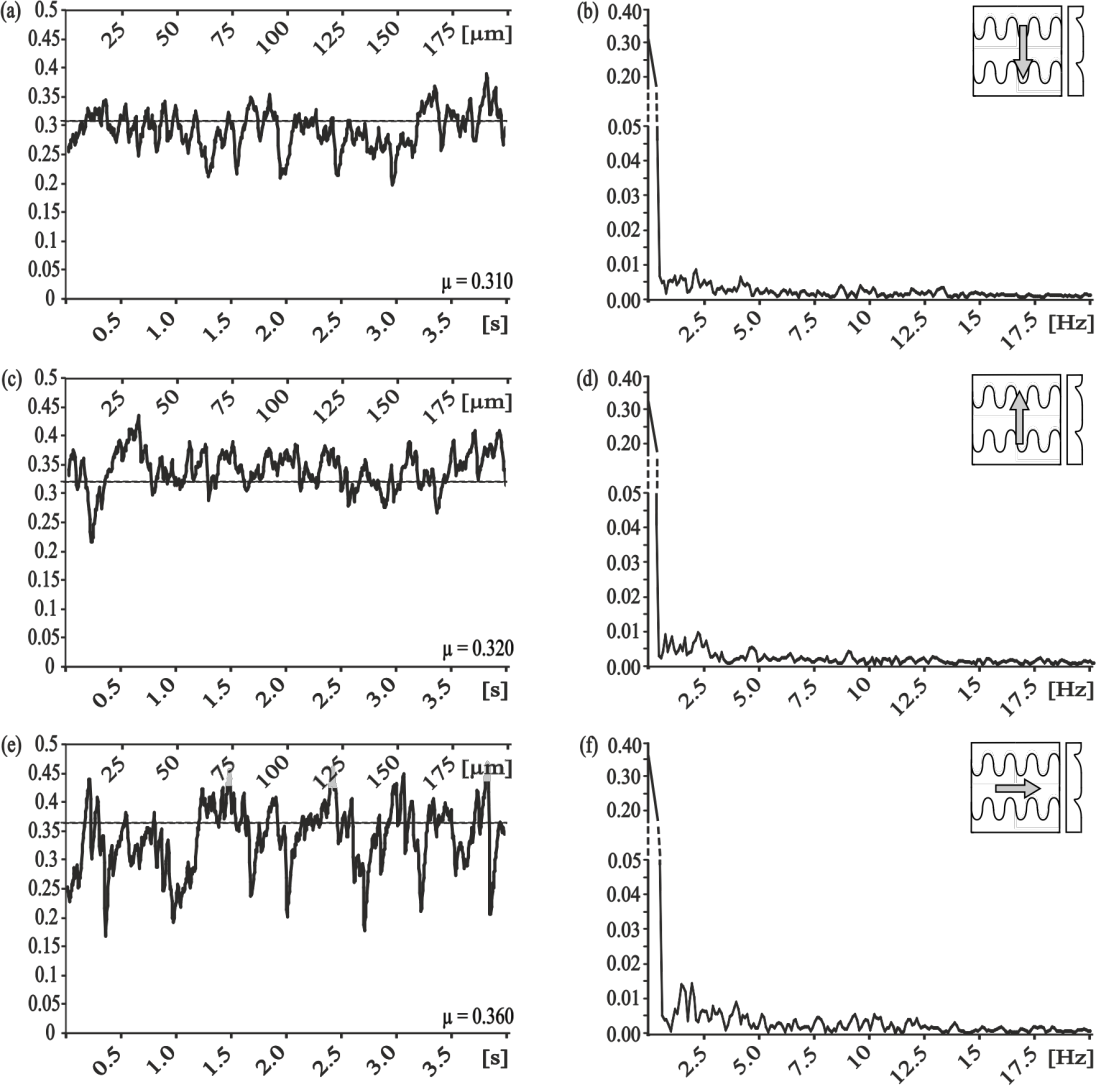
Frequency analysis of frictional coefficients measured on molds of snake skin (*L. g. californiae*) - PMLG. Left column, frictional signal in the spatial/time domain. Right column, frictional signal in the frequency domain after FFT; ordinate shows single-sided amplitude spectrum - SSAS. Measurement direction: a,b) along the microstructure, c,d) against the microstructure and e,f) lateral to the microstructure.

The frictional measurements on polymer molds from ventral scales of *L. g. californiae* (PMLG) showed anisotropic frictional properties. The measurement along the microstructure, corresponding to the forward motion of a snake, resulted in a frictional coefficient of 0.31 ± 0.02. In opposite direction, this coefficient was significantly higher (0.32 ± 0.01). Comparison of results obtained in these sliding directions to those obtained in the lateral direction demonstrated the pronounced frictional anisotropy (µ lateral: 0.36 ± 0.02). The occurrence of stick-slip behavior was minimal (maximal amplitude: <0.01) for the forward (Figure 5b) and backward (Figure 5d) directions, whereas it was slightly stronger (maximal amplitude: 0.015–0.025) (Figure 5f) for the lateral direction. For all measured directions on this type of microstructured surface, no dominant frequency was detected.

### Stick-slip behavior of snake-inspired microstructured polymer surface (SIMPS)

**Figure 6:**
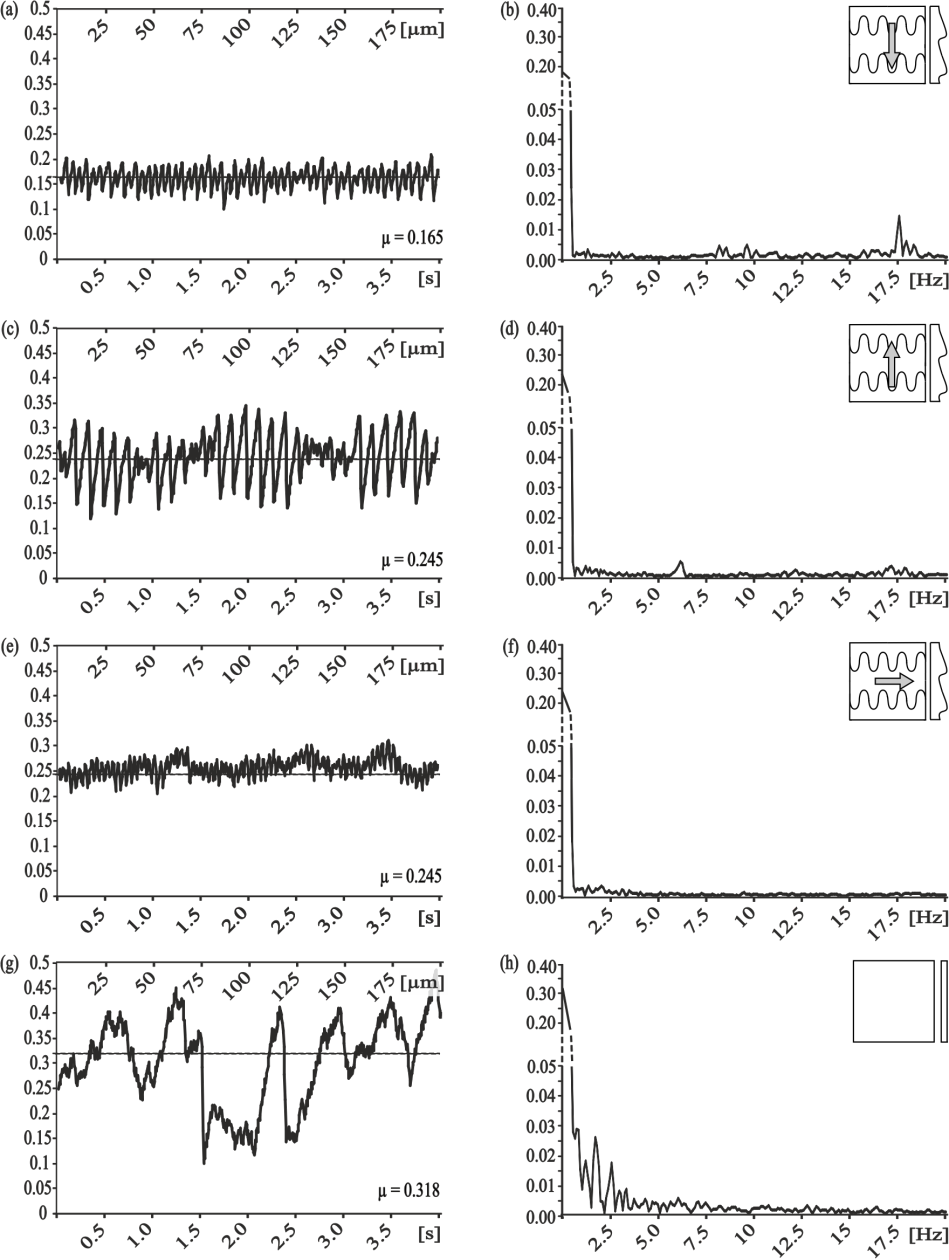
Frequency analysis of the frictional coefficient measured on snake-inspired microstructured polymer surface - SIMPS (a–f) and smooth surface (h,i). Left column, frictional signal in the spatial/time domain. Right column, frictional signal in the frequency domain after FFT; the ordinate shows the single-sided amplitude spectrum - SSAS. Measurement direction: a,b) along the microstructure, c,d) against the microstructure and e,f) lateral to the microstructure, g,h) on smooth surface.

The frictional measurements on SIMPS show a low frictional coefficient of 0.17 ± 0.00 along the microstructure and a higher one of 0.25 ± 0.01 in the opposite and in the lateral direction of 0.25 ± 0.01. The detected dominant frequency after performed FFT (Figure 6b) corresponds to a wavelength of 2.9 µm which correlates with the distance between two rows of snake-inspired finger-shaped microstructures (Figure 1c,e).

The frictional measurements on the mold of a smooth surface showed an erratic frictional behavior with a medium frictional coefficient of 0.32 ± 0.01 (Figure 6g,h).

## Discussion

We used FFT to analyze signals obtained from frictional measurements. It allowed us to gain the average frictional coefficient and information on both the dominating frequencies and amplitudes of the frictional signal. Therefore, we were able to investigate in great detail the variations in the frictional behavior depending on the surface topography.

Our data did not confirm the previous statement (e.g., [27–28]) that a low frictional coefficient correlates with no or a minimal occurrence of stick-slip behavior. The contact pairs with the lowest frictional coefficients showed in the frequency spectrum maximal amplitudes between 0.010–0.025, not minimal stick-slip amplitudes (maximal amplitude: <0.010), as expected. Substrates that showed almost no stick-slip behavior had medium to high frictional coefficients.

The stick-slip phenomenon is very important for technical surfaces under tribological stress, but only little understood and hard to quantify and control. There are many approaches to optimize frictional properties of surfaces and to affect the occurrence of stick-slip, e.g*.*, (1) wet or solid lubrication, (2) additional application of dampening polymers, (3) use of specific microstructures or (4) application of vibrations (e.g., [23,29]).

The application of small amplitude vibrations perpendicular to the sliding direction is a remarkable approach to eliminate stick-slip. This mechanical control of the frictional system is aimed at reducing the frictional coefficient and to reduce or to eliminate stick-slip motion by stabilization of the relative motion of the sliding partners [23,28–30]. Applying vibrations normal to sliding directions can modulate the real contact area between sliding partners like microstructured surfaces, meaning external vibrations lead to a "virtual" surface roughness and are thereby able to influence frictional properties in a comparable way.

Reducing friction-induced instabilities by active dampening is advantageous due to simple controllability and adaptability to changing system properties, e.g., changing temperature. The resulting reduction in stick-slip motion can be caused by the interference of the induced vibrations with the stick-slip motion of the sliding surfaces and the externally applied vibrations normal to the sliding direction.

A similar effect can be reached by the application of microstructures to the sliding surfaces. The microstructure results in a continuous and defined periodical forming and breaking of contact, which can be a source for discrete occurring oscillations and thereby specific frequencies within a frictional signal. By optimizing the dimensions of the microstructure and specific vibrations induced in this way, this could be a method to reduce stick-slip motion without the need of an external oscillator [23,27,31–33]. Due to the fact that frictional properties in general and stick-slip behavior in particular also strongly depend on material properties of the sliding partners, the optimal stick-slip reducing dimension of microstructures must be engineered for every single technical application. Nevertheless, it could be a very effective way to reduce frictional energy loss and friction-induced wear.

In the present paper, systematic investigation of the influence of different types of geometry and dimensions of the microstructure on frictional coefficients and the stick-slip behavior, we made following observations. Sliding perpendicular to the PGMS is a proper way to induce vibrations with discrete frequencies (Figure 2). Based on the fact that every dry frictional system is confronted by the stick-slip motion of sliding, flat, solid surfaces [23,25], the use of microstructures like PGMS oriented perpendicular to the sliding may be a promising way to reduce stick-slip and tune the frequency of stick-slip motion by varying the microstructure dimensions.

In order to interpret the results obtained on PGMS it is necessary to take a closer look at relative dimensions between the pitch size of the microstructure and the glass ball as the counterpart. Calculated penetration depths of the glass ball into the microstructures, based on geometry only are listed in Table 4.

**Table 4:** Penetration depths of the glass ball into the periodical groove-like polymer surface – PGMS at different pitch dimensions based on geometry only. The calculated values, which lay beyond the system’s resolution in vertical direction (cantilever deflection in vertical direction, smaller 50 nm), are shown in italics.

	PGMS - 5 µm	PGMS - 25 µm	PGMS - 50 µm	PGMS - 100 µm

Penetration depth [µm]	*0.002*	*0.039*	0.156	0.625

It is necessary to notice that the spatial resolution of the microtribometer for cantilever deflection in normal direction is too low to detect the deflection on microstructures with pitch dimensions of 5 µm and 25 µm (penetration depth: 0.002 µm and 0.039 µm, respectively, for details see Table 4). One explanation why we were nevertheless able to observe distinct frequencies in the frictional signal within these dimensions could be the periodic variation in the real contact area during sliding perpendicular to the microstructure. These periodic changes in the real contact area cause variations in critical stiction length as reported in Yu and Wang (2012) [34]. Presuming this assumption is right, we were able to detect the stiction length measured perpendicular to the microstructure on PGMS with pitch dimensions of 5 µm and 25 µm. The observed frequencies measured perpendicular to the large-scale microstructures (PGMS pitch dimensions of 50 µm and 100 µm) can be induced by a combination of the interlocking phenomenon between the frictional partners and the variation of real contact area.

Sliding along the PGMS shows a more chaotic frictional behavior with no distinct single frequencies within the frictional signal. Statistic analysis of these data showed only a significant difference in between the biggest and the smallest pitch dimensions (p < 0.001, one way ANOVA followed by the Holm–Sidak method).

Dealing with the results of friction measurements perpendicular and along the microstructures, one could assume that the influence of the interlocking phenomenon on the frictional coefficient is rising with increasing pitch dimensions. However, our experimental setup showed that reduced stick-slip motion does not mandatorily correlate with a low frictional coefficient. For example, frictional measurements parallel to the small-sized microstructure showed very little stick-slip motion, but a relatively high frictional coefficient (Figure 3a–d). Bigger dimensions of the microstructure caused a decrease of the frictional coefficient, but an increase in the amplitude of the stick-slip-induced vibrations (Figure 3g,h).

Frictional measurements on RRS showed similar results regarding the non-discrete frequencies within the frictional signal. Consistent with the results for the PGMS, the lowest frictional coefficient did not correlate with a minimal stick-slip motion. Results for frictional measurements on polymeric molds from a living snake (PGLG) showed comparatively high frictional coefficients independent of measurement directions, but none the less, evident frictional anisotropy. Compared to other measured surfaces mentioned above, the stick-slip motion was reduced here, and the frictional coefficients increased. We concluded from these findings, that the anisotropic frictional properties of snake skin [17] cannot be simply be copied by producing polymeric replicas of the original snake surface. The frictional coefficient of snake skin is not only influenced by the surface microstructure but by multiple parameters [17,35]. Frictional measurements on SIMPS showed anisotropic frictional properties and quite different types of stick-slip behavior (Figure 6) in the measurements along the microstructure, showing the lowest frictional coefficient and moderate occurrence of stick-slip. The similarity in the reduction of stick-slip-motion in PMLG and SIMPS led us to conclude, that this physical phenomenon is influenced by surface microstructures. The cause for this effect is possibly the periodicity of the microstructure, which can influence the critical stiction length during sliding, as reported by Yu and Wang (2012) [34]. Due to the fact, that snake skin has to fulfill multiple functions it can be assumed, that the tribological optimization is related to multiple physical phenomena, meaning the surface modifications bring frictional optimization [17], reduction of wear rate [35] and as shown in our study at hand reduction in stick-slip motion with it. The reduction of stick-slip behavior is directly related to the reduction of wear. Additionally, "controlled" stick-slip motion compared to "uncontrolled", randomly appearing stick-slip makes the occurring forces within a frictional system more predictable and prevents the occurrence of maximum forces, which could damage the system [31,33]. Therefore the reduction in stick-slip behavior can help to maintain optimal frictional properties of a tribological system.

To investigate the correlation between frictional coefficient and stick-slip behavior, the data were grouped into (i) low frictional coefficient and (ii) reduced stick-slip motion. The group (i) included the following samples under particular experimental conditions: PGMS, λ = 25 µm, perpendicular to the microstructure (µ = 0.167 ± 0.008); PGMS, λ = 100 µm, parallel to the microstructure (µ = 0.172 ± 0.024); RRS - 9 µm (µ = 0.192 ± 0.007); and SIMPS, along the microstructure (µ = 0.165 ± 0.010). The frequency analysis of frictional measurements on these microstructured surfaces showed frequencies of medium amplitude (maximal amplitude: 0.015–0.025).

The group (ii) of samples with reduced stick-slip motion was subdivided depending on the maximal amplitude into two groups, where stick-slip motion was (a) nearly eliminated (maximal amplitude: <0.005) and (b) strongly reduced (amplitude <0.015). Members of the first group were SIMPS measured in lateral direction and PGMS with pitch dimension of 5 µm measured parallel to the microstructure. For both samples and experimental setups, the amplitudes of the stick-slip frequencies were smaller than 0.005, and the frictional coefficients were relatively high, 0.250 ± 0.018 and 0.228 ± 0.016, respectively. The second group with reduced stick-slip motion contained the following surfaces: PGMS with pitch dimensions of 25 µm and 50 µm measured parallel to the microstructure (µ = 0.196 ± 0.011, µ = 0.198 ± 0.022, respectively); PGMS with pitch dimension of 5 µm measured perpendicular to the microstructure (µ = 0.290 ± 0.006); RRS - 0.3 µm (µ = 0.284 ± 0.027) and RRS - 1 µm (µ = 0.264 ± 0.008); molds of the snake along (µ = 0.31 ± 0.018), against (µ = 0.32 ± 0.009), and lateral (µ = 0.361 ± 0.016) to the microstructure; and SIMPS along (µ = 0.165 ± 0.010), against (µ = 0.245 ± 0.019), and lateral (µ = 0.250 ± 0.018) to the microstructure.

Following the accepted assumption of a correlation between stick-slip motion and frictional coefficient (e.g., [27–28]), one would expect to find low frictional coefficient accompanied by reduced stick-slip motion, but we were not able to confirm this expectation for frictional systems examined. One explanation of this could be the fact that most experiments on solid frictional systems were performed on metal-metal contact pair (e.g., [15,23,25]) and thereby differ in their physical properties very much from polymeric systems under consideration.

In the previous study, we focused on the effect of the effective elastic modulus on friction and thereby on the dampening within the frictional system snake-substrate [17]. We investigated the effect by measuring friction of snake skin with and without soft cushioning. It was assumed that the ability of the snakes to vary the effective elastic modulus by varying their body stiffness is useful to optimize their tribological properties in adaptation to different substrates and locomotion modes [2,36–37]. Given that the effective elastic modulus is varied in a highly optimized frictional system like snakes and taking the present data into account, the dampening has a big influence on the occurrence of stick-slip phenomenon during sliding. It is postulated that the amplitude of friction-induced vibrations is dependent on the system's dampening (e.g., [23,29]). The investigation of the effect of dampening on the frictional coefficient and stick-slip motion in biological surfaces may represent a short cut towards frictional systems with reduced stick-slip.

## Material and methods

### Surface fabrication and characterization

**Figure 7:**
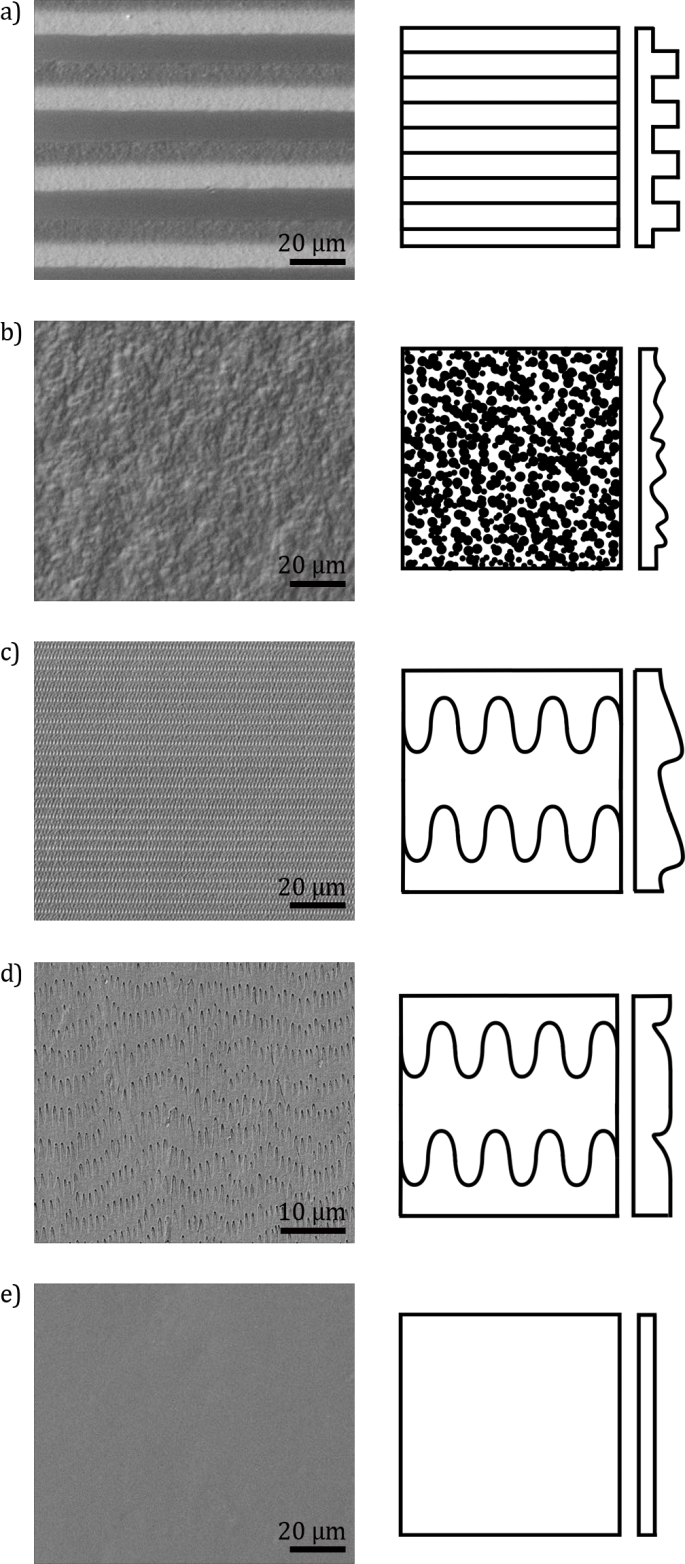
Exemplary overview of the topography of the examined polymer surfaces. Left column: SEM-micrographs. Right column: schematic top view and profile of the surface microstructure. a) Periodical groove-like polymer surface – PGMS (pitch: 25 µm). b) Randomly-rough surfaces – RRS (grain size: 1 µm). c) Snake-inspired microstructured polymer surface – SIMPS. d) Mold of the ventral scale of *L. g. californiae* - PMLG. e) Smooth surface.

Following types of surface topography were masters: (i) a periodically groove-like microstructured polymer surface (PGMS), (ii) randomly rough surfaces (RRS) with grain sizes of 0.3, 1, 3, 9 and 12 µm (FibrMet Discs, Buehler GmbH, Düsseldorf, Germany), (iii) polymeric molds of ventral scales of *L. g. californiae* (PMLG) and (iv) a snake inspired polymer surface (SIMPS), produced in cooperation with the Leonhard Kurz Group Stiftung & Co (Fürth, Germany) and (v) a smooth glass surface as reference (Figure 7). Experiments on molds of the ventral snake surface and on SIMPS were conducted to reveal the performances of snake-inspired microstructures. The PGMS samples, characterized by a rectangular profile in cross section with four different structural wavelengths of 5, 25, 50 and 100 µm containing equal ridges and grooves (produced by the Laser Zentrum Hannover e. V., Hannover, Germany). Those were taken for the purpose of investigating the scale effects of the microstructure. Additionally, by sliding along or across the microstructure, it was possible to investigate the influence of an anisotropic geometry of microstructures on frictional properties. The RRS samples with different grain sizes were used to investigate the influence of the surface roughness, the contact area, and the interlocking of surface asperities.

The detailed values for surface roughness are listed in Table 1. The polymer surfaces were produced by a two-step molding technique [38]. In the first step, the master surface was covered with fluid two-component polyvinylsiloxane (PVS) (Coltène President light body, Coltène Whaledent Dentalvertriebs Ltd., Constance, Germany), which polymerizes within few minutes. In the subsequent second step, the negative cast was filled with the low-viscosity epoxy resin [19]. This polymer (Polysciences Inc., Eppelheim, Germany) consists of (1) nonenyl succinic anhydride (NSA) (61.3%), (2) 3,4-epoxycyclohexylmethyl-3,4-epoxycyclohexyl-carboxylate (ERL 4221) (23.6%), (3) diglycidyl ether of polypropyleneglycol (D.E.R. 736) (14.2%), and (4) *N*,*N*-dimethylaminoethanol (DMAE) (0.9%). The polymerization of the resin took place over night at 70 °C.

The surface roughness (*R*_a_) of molds was measured with a white light interferometer (New View 6000, ZygoLOT, Darmstadt, Germany). For metrologic characterization of the surface microstructures, the image analysis software SigmaScanPro 5.0 (SPSS Inc., Chicago, USA) was used. Roughness (*R*_a_) perpendicular to the lines of the PGMS samples was calculated depending on the pitch width and depth.

For surface visualization, two scanning electron microscopes (SEM) were used (Hitachi S-4800 and TM-3000, Hitachi High-Technologies Corporation, Tokyo, Japan) at an acceleration voltage of 2–3 kV and 5 kV, respectively. The polymer material was fixed by adhesive pad and sputter-coated with gold/palladium (80:20) with a layer thickness of 20 nm. For sputter-coating, the high vacuum sputter coater Leica EM SCD500 (Leica Microsystems GmbH, Wetzlar, Germany) was used.

Detailed characterization of the surface topography was performed by a NanoWizard® atomic force microscope (JPK Instruments), mounted on an inverted light microscope (Zeiss Axiovert 135, Carl Zeiss MicroImaging GmbH). The SIMPS were imaged by using the intermittent contact mode of the AFM. The error channel (also known as the amplitude channel) visualizes the change in damping of the cantilever amplitude while scanning the surface. Only images obtained with the error channel are shown, because this visualization method is helpful to gain a more vivid imaging of the surface topography. Scans were carried out at a 1 Hz scan rate and a resolution of 1024 × 1024 pixels with an intermittent contact mode cantilever (*c* = 50 Nm^−1^, NST-NCHF, Nascatec GmbH, Stuttgart, Germany), at ambient conditions (room temperature 24 °C, relative humidity 41%). NanoWizard® SPM software 3.3.23 (JPK Instruments) was used to obtain AFM images and NanoWizard® image processing software 3.3.25 was applied to extract 3D surface profiles.

### Frictional measurement

Frictional measurements were carried out with the microtribometer Basalt-MUST (TETRA GmbH, Ilmenau, Germany). 2-dimensional force detection was accomplished by a metal cantilever (*C*_Fn_: 22.3 N/m, *C*_Ft_: 23.1 N/m). The spacial resolution of the system in detection a deflection of the cantilever was 50 nm. The averaged applied normal force was 0.6 mN. The measurement was performed over a sliding distance of 500 µm at a velocity of 50 µm/s. As contact partner, a glass ball with a diameter of 1 mm was chosen and fixed to the cantilever by cyanoacrylate glue (Ergo 5925 Elastomer, Tagelswangen, Switzerland). The roughness of the glass ball determined by a white light interferometer (NewView, ZygoLOT GmbH, Darmstadt, Germany) was *R*_a_ = 0.006 µm. The polymer surfaces were fixed on metallic sample holders by cyanoacrylate glue.

**Figure 8:**
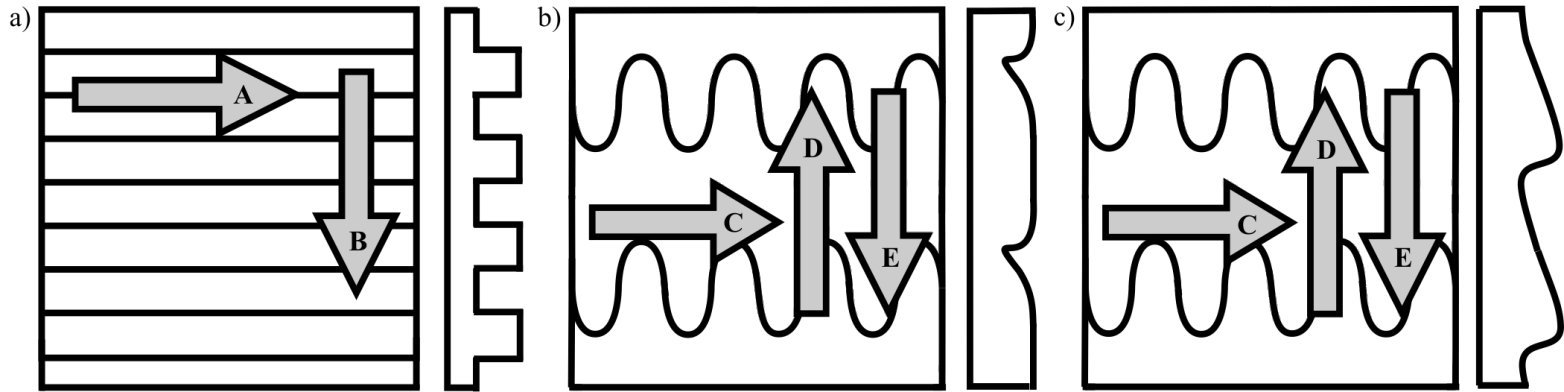
Scheme of surface geometry and sliding directions of friction measurement, top view (left) and side view (right). a) Periodical groove-like polymer surface - PGMS, b) Polymeric mould of *L. g. californiae –* PMLG, c) Snake-inspired microstructured polymer surface – SIMPS. Directions of measurement: A: parallel to microstructure, B: perpendicular to microstructure, C: lateral to microstructure, D: against microstructure, E: along microstructure.

To characterize frictional properties of the periodic groove-like patterned surfaces (PGMS) friction measurements in two different directions were done: parallel to the microstructure (i) and perpendicular to the microstructure (ii) (Figure 8a). For frictional characterization frictional measurements on polymeric moulds of *L. g. californiae* (PMLG) were executed in four different directions: along the microstructure (i), corresponding to the forward movement of the animal, against the microstructure (ii), corresponding to the opposite direction, and lateral direction relative to the body axis (Figure 8b). The frictional properties of the SIMPS, was measured in three different directions: along the anisotropic microstructure (i), against the anisotropic microstructure (ii), and in the lateral direction, perpendicular to both other directions (iii) (Figure 8c). For the characterization of surfaces with isotropic topography (RRS and smooth surface), frictional measurements were done in one directions.

Individual measurements were repeated 15 times on each SIMPS and on the smooth reference surface. For the molds of the snake scales three frictional measurements on three different scales of three different individuals were performed. The other surfaces were tested five times each. Each measurement was performed on a new area of the surface to minimize the influence of abrasion. Obtained data were statistically analyzed with SigmaPlot 11.0 software (SPSS Inc., Chicago, USA). Kruskal-Wallis one way ANOVAs followed by Holm–Sidak tests with a significance level of p < 0.05 were performed.

The contact area was calculated by the Hertz-model [39] using the data on E-moduli of the polymerized Spurr resin and the glass ball (7 GPa and 70 GPa, respectively [40]) and an assumed Poisson ratio of 0.5 for both materials. The calculation of the contact area (Hertz-model) between non-microstructured polymer surface and smooth glass ball gave an apparent area of 40 µm^2^.

To characterize the frictional properties of the surfaces with microstructures with anisotropic geometry, the measurements were performed in different sliding directions: along, against, and lateral to the microstructures, as illustrated in Figure 8.

### Data processing

The stick-slip behavior of microstructured polymer surfaces was characterized by the analysis of the frequency spectrum gained by fast Fourier transformation (FFT) of the frictional signal (of the spatial domain). In general, the FFT is used to transform a function of time into a function of frequency. In the frequency spectrum, the temporal domain of a sinusoidal signal can be visualized in the spatial domain. By performing an inverse FFT, the original signal can be reproduced. Until now, most investigations on the stick-slip phenomenon are based on the simple consideration of the frictional coefficient over the measured distance or time (e.g., [20–21,26–28,33,41–43]). However, this technique is not sufficient to identify dominant frequencies, when several sinusoidal oscillations are overlapping each other. In this case, only a detailed analysis of the frequency spectrum can identify all present frequencies and their corresponding amplitudes. By using this approach for data analysis, we were able to identify friction induced vibrations caused by the stick-slip phenomenon as well as the frictional coefficient with the same mathematical tool. Within the frequency spectrum, the mean friction coefficient and frequencies of the dominant friction-induced vibrations can be identified.

**Figure 9:**
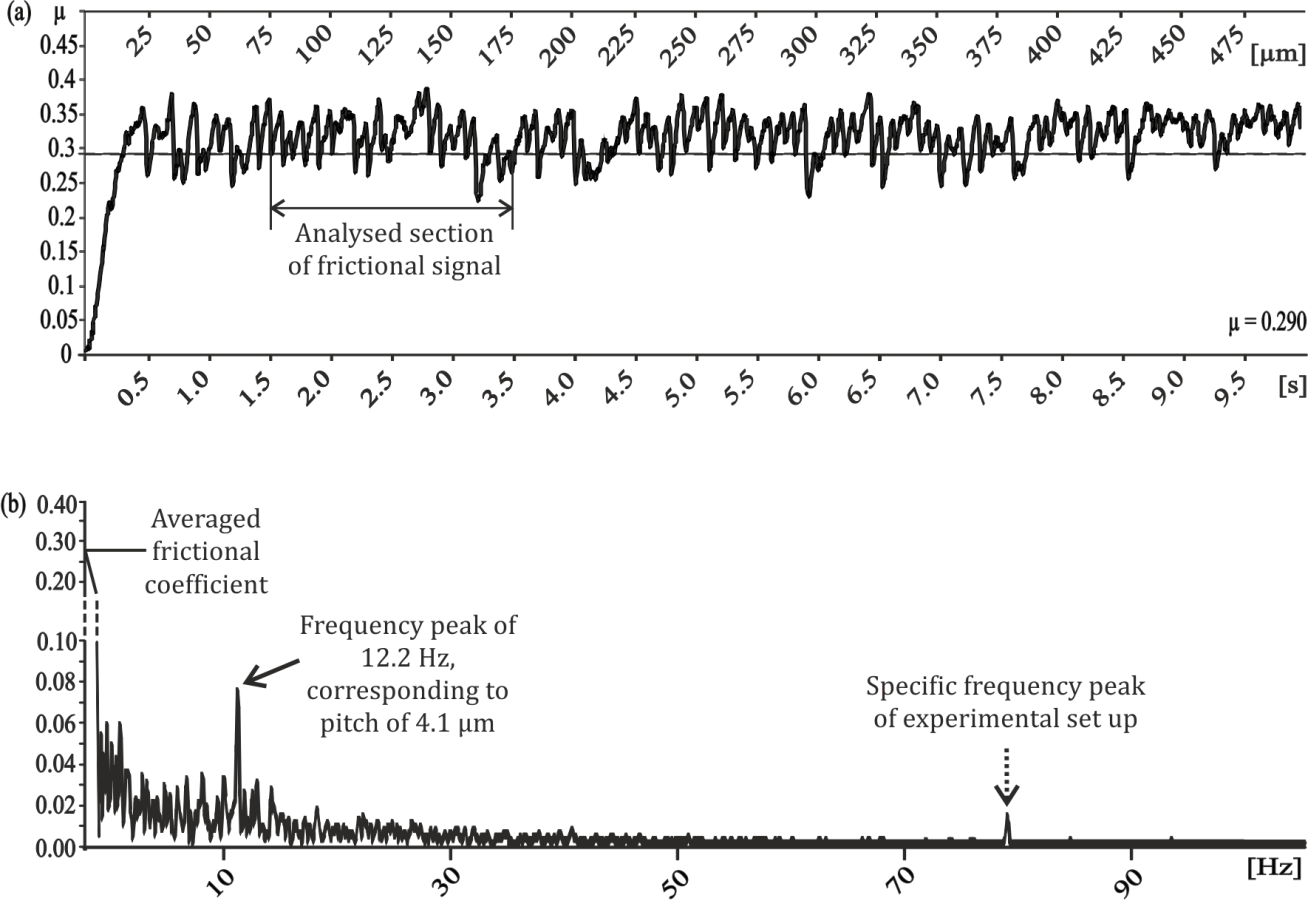
Example of data analysis of the frictional signal measured on the periodical groove-like polymer surface – PGMS (pitch dimension: 5 µm) perpendicular to the orientation of the microstructure, a) in spacial/time domain. Averaged frictional coefficient and exemplary selected section of frictional signal for data processing; b) in frequency domain based on FFT, ordinate shows single-sided amplitude spectrum – SSAS. Intersection of the graph on the ordinate presents the averaged frictional coefficient. At 12.2 Hz a dominating frequency according to a pitch of 4.1 µm can be identified. This correlates well to the pitch of 5 µm (lines and spaces of the substrate). Dotted arrow points out the resonance frequency peak, which is the property of the experimental setup, because it could be found in each combination of tribometer, glass ball, and epoxy mold samples.

In detail, the following procedure was applied to analyze friction behavior, exemplarily shown in form of a typical friction curve with the friction coefficient (defined according to Coulomb’s friction law µ = Ft/Fn) over time or displacement, respectively (Figure 9). The exemplary data results from the experiment, where a glass sphere moved perpendicular to the microstructure of a PGMS with a pitch size of 5 µm. One section of the data, typically representing a distance of about 100 µm and exhibiting dynamic friction (without static friction and obvious disturbances) was chosen for further processing (Figure 9a). The data set was then Fourier transformed after zero padding to the next power of 2 and weighting by a Hamming window. Data processing was performed using the build-in *fft*-command of MATLAB Version 7.12.0.635 (R2011a) (MathWorks Inc., Ismaning, Germany). For the sake of convenience, we only show the single-sided amplitude spectrum (SSAS). Figure 9b shows the SSAS of the data section shown in Figure 9a. In general, the zero frequency peak is the off-set or bias of the friction curve and can be identified as the average friction coefficient. Furthermore, a characteristic peak at 79.3 Hz was found in all measurements and is attributed to the resonance frequency of the experimental setup (glass ball fixed on a metal cantilever in contact with the surface). Since no measurement frequencies higher than 20 Hz were obtained, except for the resonance frequency, the SSAS is cut at that frequency. Whenever a periodic friction behavior was observed, e.g., induced by the periodic microstructure, a peak at a specific frequency or wavelength was detected in the SSAS. Exemplary, in Figure 9b, a peak at about 12.2 Hz, corresponding to a wavelength of about 4.1 µm at the deployed speed of 50 µm/s, is revealed. Since this correlates well with the pitch of 5 µm of the PGMS surface, we are confident with our procedure.
